# Therapeutic effect of fecal microbiota transplantation on chronic unpredictable mild stress-induced depression

**DOI:** 10.3389/fcimb.2022.900652

**Published:** 2022-07-28

**Authors:** Ting Cai, Shao-peng Zheng, Xiao Shi, Ling-zhi Yuan, Hai Hu, Bai Zhou, Shi-lang Xiao, Fen Wang

**Affiliations:** ^1^ Department of Gastroenterology, The Third Xiangya Hospital, Central South University, Changsha, China; ^2^ Hunan Key Laboratory of Nonresolving Inflammation and Cancer, The Third Xiangya Hospital, Central South University, Changsha, China; ^3^ Department of Orthopedics, The Third Xiangya Hospital, Central South University, Changsha, China

**Keywords:** depression, fecal microbiota transplantation, intestinal microflora, gastrointestinal mobility, metabolites

## Abstract

**Background and objective:**

Depression is a complex neuropsychiatric disease with extensive morbidity. Its pathogenesis remains unclear, and it is associated with extremely low rates of cure and complete remission. It is vital to study the pathogenesis of depression to develop effective treatments. This study aimed to explore the therapeutic effects and mechanisms of fecal microbiota transplantation (FMT) for the treatment of depression in rats.

**Methods:**

Thirty Sprague-Dawley (SD) rats were randomly divided into three groups: control, chronic unpredictable mild stress (CUMS) to model depression, and CUMS+FMT. For the CUMS and CUMS+FMT groups, after CUMS intervention (four weeks), the rats were given normal saline or FMT (once/week for three weeks), respectively. Behavior, colonic motility, 16S rDNA amplicon sequencing, and untargeted metabolomics on fecal samples were compared between the three rat groups. The following markers were analyzed: 5-hydroxytryptamine (5-HT), gamma-aminobutyric acid (GABA), glutamate (Glu), and brain-derived neurotrophic factor (BDNF) levels in the hippocampus; glucagon-like peptide 1 (GLP-1), lipopolysaccharide (LPS), and interleukin (IL)-6 levels in the serum; and GLP-1, GLP-1 receptor (GLP-1R), and serotonin 4 receptor (5-HT_4_R) levels in colonic tissues.

**Results:**

FMT improved symptoms of depression and colonic motility in rats exposed to CUMS. The expression levels of 5-HT, GABA, BDNF, and other biochemical indices, significantly differed among the three groups. Meanwhile, the intestinal microbiota in the CUMS+FMT group was more similar to that of the control group with a total of 13 different fecal metabolites.

**Conclusion:**

FMT exerted antidepressant effects on CUMS-induced depression in rats, and the mechanism involved various neurotransmitters, inflammatory factors, neurotrophic factors, and glucagon-like peptides.

## Introduction

Depression is a complex neuropsychiatric disease with extensive morbidity. The global incidence of depression has been reported as high as 4.4% (Tran et al., 2020). The pathogenesis of depression is still unclear, treatment methods are limited, and complete remission or cure rates for depression are very low (Malhi and Mann, 2018). Therefore, it is critical to obtain an understanding of the pathogenesis of and effective treatment for depression. FMT aims to treat certain diseases by restoring or reconstructing the intestinal microbiota of patients by transplanting the intestinal microbiota of healthy persons to the intestines of patients. Currently, FMT has achieved positive results for the treatment of recurrent Clostridium difficile infection. Some studies have reported the application potential of FMT in neuropsychiatric diseases such as multiple sclerosis (Makkawi et al., 2018), autism (Bibbò et al., 2017), and epilepsy (He et al., 2017). An increasing body of evidence exists demonstrating the close association of the intestinal microbiota and depression (Jianguo et al., 2019; Li et al., 2019). However, there is a lack of evidence regarding the effects of FMT on depression. In the early stages of this research, our research group successfully used FMT to cure a patient with depression caused by a stressful life event (Cai et al., 2019). This study revealed a disordered intestinal microbiota in patients. FMT led to an alteration in the intestinal microbiota and an improvement in depressive symptoms, constipation, and abdominal distension. The current study aims to explore the possible mechanism of intestinal microbiota composition for the treatment of depression through the establishment of an animal model of depression with FMT intervention.

## Materials and methods

### Experimental animals and grouping

Thirty healthy male specific-pathogen-free SD rats (body mass, 180-220 g) were provided by the Hunan SJA Laboratory Animal Co. Ltd. (quality certificate number of experimental animals: 43004700050241). The animals were kept in the Department of Laboratory Animals of the Central South University. During the acclimation period, the rats were housed with a 12 h light/dark cycle (lights on from 08:00–20:00), constant temperature of 23 ± 1°C, and relative humidity of 55–65%, and were provided food and water ad libitum. After seven days of adaptive feeding, all rats were randomly divided into one of three groups (10 rats/group): the control group (two rats/cage), the CUMS group (one rat/cage), and the CUMS+FMT group (one rat/cage). All procedures were in accordance with the Standards for the Use of Laboratory Animals issued by the Ministry of Health of the People’s Republic of China (Order No. 55 of January 25, 1998), and approved by the Committee for the Protection and Use of Laboratory Animals of the Central South University.

### Experimental intervention

In the control group, rats were housed in a normal fashion every day without any stimulation. In the CUMS and CUMS+FMT groups, the rats were subjected to one of the following stressors every day in a random order: water deprivation for 24 h, food deprivation for 24 h, tail clamp for 1 min, restraint for 1 h, cage exchange for 24 h, humid environment for 24 h, and overnight lighting for 36 h. After modeling for four weeks, the rats were gavaged once per week for a total of three weeks. Rats in the CUMS+FMT group received a gavage with fecal bacterial fluid from the normal group (1 mL/100 g body weight) and the CUMS group was gavaged with normal saline (1 mL/100 g body weight). Chronic stress stimulation continued until the end of the last gavage.

### Detection of experimental indices

Body weight measurement: The body weight of each rat was measured at 8 a.m. every Tuesday during the experimental period.

Food intake detection: The food intake of all rats was measured for 24 h before and after modeling and after the third gavage.

Sucrose preference test: For the sucrose preference test, each rat was provided with one bottle of tap water and another bottle containing 1% sucrose solution for 1 h, and the amounts of sucrose solution and water consumed were recorded. The sucrose preference rate was calculated as sucrose consumption (g)/(sucrose consumption [g] + water consumption [g]). To avoid neophobia, the rats were first exposed to the 1% sucrose solution for 24 h before the sucrose preference test was carried out.

Open-field test: The open-field apparatus consisted of a 100 × 100 cm square arena with 35-cm-high walls made of black aluminum alloy board. The test was conducted after modeling and after the third gavage. The total distance, resting time, and number of times the rats were upright were recorded for 5 minutes for rats in all the groups.

Forced swimming test: A forced swimming test was conducted after the modeling and after the third gavage. For this, the rats were successively placed in a plexiglass cylinder (60 cm, height; 20 cm, diameter) filled with water (24°C ± 2°C) to 30 cm, and the time of immobility was recorded for 5 min.

Glass bead discharge test: After the third gavage, the rats were subjected to water and food distal deprivation for 12 h. For the glass bead discharge test, 3 mm glass beads were pushed 3 cm into the colon of the rats, and the time required for each rat to expel the glass bead (3mm, diameter) was recorded.

16s rDNA amplicon sequencing and untargeted metabolomics analysis: After modeling and at four days after the third gavage, rat feces from all three groups were collected in disposable sterile specimen tubes and frozen at –80°C until further analysis. 16S rDNA sequencing and metabolomics analysis were carried out by GeneTalks and Shanghai Applied Protein Technology Co. Ltd.

Immunohistochemistry: Brain tissue samples were subjected to immunohistochemical analysis to detect levels of 5-hydroxytryptamine (5-HT), gamma-aminobutyric acid (GABA), glutamate (Glu), and BDNF. Paraffin-embedded samples were cut into 4-µm-thick sections and incubated with the corresponding antibodies (Wuhan Servicebio Technology Co. Ltd., Wuhan, China) at 4°C overnight, followed by incubation with the corresponding secondary antibody (Wuhan Servicebio Technology Co. Ltd., Wuhan, China) for 30 min. They were then stained with 3,3′-diaminobenzidine (DAB; Wuhan Servicebio Technology Co. Ltd., Wuhan, China). The scoring was based on the positive area proportion and immunostaining intensity: Cells without staining were negative, and scored as 0; weakly positive cells that stained light yellow were scored as 1 point; medium yellow or brown cells with no background staining, or medium positive cells with dark brown but light brown background, 2 points; strongly positive cells with dark brown or tan color and no background staining, 3 points. Samples were scored according proportion of positive area as follows: 1–10% positive were scored as 1; 11–50% positive, 2; 51–80% positive, 3; and>80% positive, 4. Five different (×400) fields in the hippocampal CA1 region were randomly selected for interpretation, and the scores of the above two indices were added together: 0: (–), 1–4:(+), 5–8:(++), and 9–12:(+++).

Enzyme-linked immunosorbent assay (ELISA): Serum levels of glucagon-like peptide 1 (GLP-1), lipopolysaccharide (LPS), and interleukin (IL)-6 were detected using ELISA (ELISA kit; reagents from Cusabio Biotech Co. Ltd., Wuhan, China) according to the manufacturer’s recommendations.

Polymerase chain reaction (PCR): GLP-1 and glucagon-like peptide 1 receptor (GLP-1R) gene expression levels in colonic tissues were detected by quantitative PCR. Total RNA was extracted and reverse transcribed to cDNA, which was then amplified by qPCR for detecting the mRNA levels of targeted genes. The primer sequences used in RT-PCR were as follows: GLP-1 (Forward: 5′- GTGACCCCACTGCCCTT-3′; Reverse: 5′-CTGACCCAGCCTCTCCAC-3′) and GLP-1R (Forward: 5′- GGTCTTGAACCTCCCCTT-3′; Reverse: 5′-CCCTTTGTGGTGCTGTG-3′). The results were quantitated using the 2^–ΔΔ^Cq method, and *GAPDH* mRNA expression was used as an internal reference.

Western blot: Total protein in colonic tissues was extracted with radioimmunoprecipitation assay (RIPA) buffer containing phosphatase and protease inhibitors. The protein concentration was determined using the BCA Protein Assay kit (Beyotime, Shanghai, China). After quantification, the proteins were separated by 10% sodium dodecyl sulfate-polyacrylamide gel electrophoresis (SDS-PAGE) and transferred to polyvinylidene difluoride (PVDF) membranes (Merck KGaA, Darmstadt, Germany). Membranes were blocked with 5% nonfat dried milk, immunoblotted with a GAPDH polyclonal Ab (1:5,000), GLP-1R rabbit polyclonal Ab (1:1,000), serotonin 4 receptor (5-HT_4_R) rabbit polyclonal Ab (1:1,000) at 4°C overnight, incubated with secondary antibodies for 1 h at 37°C, and then developed with an enhanced chemiluminescence (ECL) detection system (Bio-Rad Laboratories, Inc., Hercules, CA, USA).

### Statistical analysis

Statistical Package for Social Sciences (SPSS version 22.0) was used for data analysis and measurement data were represented as mean ± standard deviation. Data compliance with normal distribution was tested using SPSS software, and Levene’s test was used to detect homogeneity of variance. Comparisons between multiple groups of data were analyzed by one-way analysis of variance. In the case of homogeneous variance, pairwise comparisons between groups were performed using Fisher’s least significance difference method. Tamhane’s T2 method was used when variance was uneven, and values of a = 0.05 and *p* < 0.05 were considered statistically significant.

## Results

### Successful construction of the CUMS depression model and effects of FMT

#### Changes in body weight

**Figure 1:**
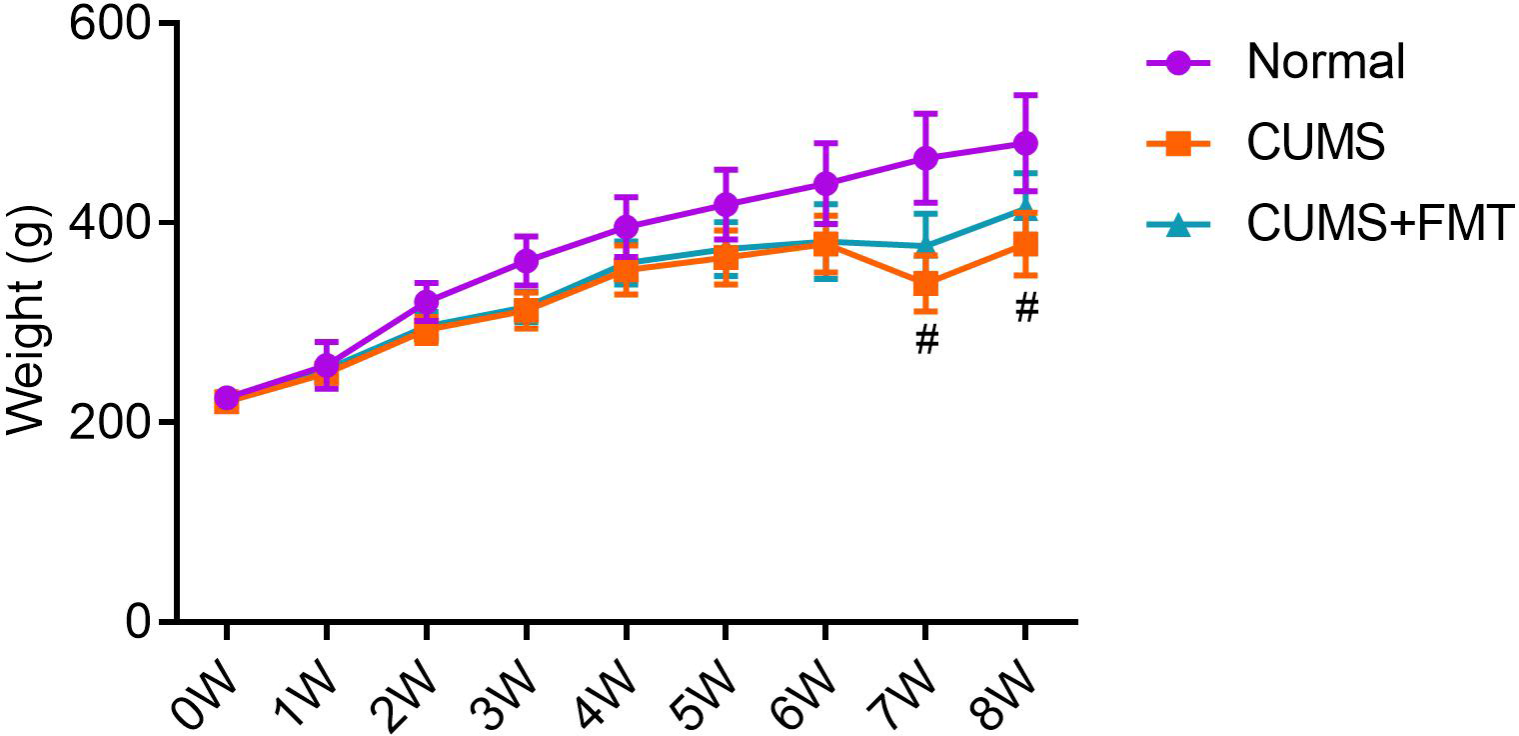
Mean weight of each experimental group throughout the study period. ^#^ *p* < 0.05 compared to CUMS group. CUMS, chronic unpredictable mild stress; FMT, fecal microbiota transplantation.

There was no significant difference in the body weight among the three groups before modeling. Within two weeks of modeling, rats in the CUMS and CUMS+FMT groups showed significant weight loss relative to rats in the control group (**Table 1**, *p* < 0.001 and *p* < 0.05, respectively). Similarly, rats in the two CUMS groups also weighed significantly less than rats in the control group at weeks three and four. During the FMT stage, food deprivation was more frequently observed at weeks seven and eight as the rats were still being subjected to stressors for CUMS, and stress stimuli were selected using the random number method. After FMT, the weights of the rats in the CUMS and CUMS+FMT groups were still lower than those of the rats in the control group. However, the rats in the CUMS+FMT group recovered some of the weight following FMT and weighed significantly more than the rats in the CUMS group (**Table 1**, **Figure 1**, *p* < 0.05).

**Table 1:** Weight comparisons at different time points in each group.

Timeline (week)	Normal	CUMS	CUMS+FMT
0	224.92 ± 8.53	220.86 ± 4.55	221.2 ± 6.38
1	257.25 ± 22.22	249.04 ± 8.67	253.24 ± 9.90
2	320.57 ± 18.34	292.85 ± 12.39***	296.18 ± 13.65*
3	361.93 ± 23.43	312.19 ± 16.95***	315.53 ± 14.40***
4	395.55 ± 28.51	352.63 ± 23.31**	359.74 ± 20.55**
5	418.22 ± 33.15	365.27 ± 25.70**	373.88 ± 25.50**
6	439.12 ± 38.74	378.57 ± 27.30**	381.03 ± 35.66**
7	464.77 ± 42.35	339.32 ± 26.56***	376.89 ± 31.15***
8	479.75 ± 45.60	378.52 ± 30.01***	414.36 ± 33.57**

Data are represented as mean ± standard deviation, n = 10/group. *p < 0.05, **p < 0.01, ***p < 0.001 compared to the control (normal) group. CUMS, chronic unpredictable mild stress; FMT, fecal microbiota transplantation.

#### Food intake

**Figure 2:**
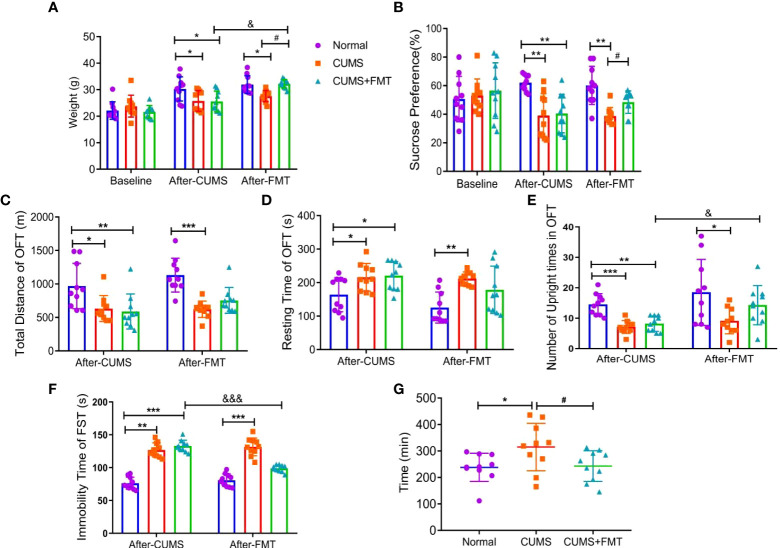
Results of behavioral experiments and glass bead discharge test. **(A)** Changes in the food intake in each group; **(B)** changes in the sucrose preference test in each group. C–E, Open-field test: **(C)** total distance; **(D)** immobility time; **(E)** Number of upright times. **(F)** Forced swimming test; **(G)** glass bead discharge test. *p < 0.05, **p < 0.01, ***p < 0.001 compared with the control group; #*p* < 0.05 compared with the CUMS group; and &*p* < 0.05, &&&*p* < 0.001 represent statistically significant differences between the values before and after FMT. Abbreviations: CUMS, chronic unpredictable mild stress; FMT, fecal microbiota transplantation.

At baseline, prior to CUMS modeling, there was no significant difference in food intake among the three groups. Four weeks after the initiation of CUMS, rats in both CUMS groups showed significantly lower food consumption than rats in the control group (*p* < 0.05). Rats in the CUMS group continued to have significantly lower food intake compared to rats in the control group for several weeks after the animals were gavaged with normal saline (*p* < 0.05). However, after FMT intervention, the CUMS+FMT group showed a significant increase in food intake compared with before FMT and CUMS group (*p* < 0.05; **Figure 2A**).

#### Sucrose preference test

At baseline, before the initiation of CUMS, there was no significant difference in sucrose consumption among the three groups. After the rats experienced CUMS to model depression, the two CUMS groups showed a significantly lower preference coefficient for sugar water (*p* < 0.01), revealing that the modeling effect was satisfactory. After FMT intervention, the preference coefficient for sugar water significantly increased in the CUMS+FMT group relative to the CUMS group (*p* < 0.05; **Figure 2B**).

#### Open-field test

After CUMS, the total distance traveled by rats in the control, CUMS, and CUMS+FMT groups were as follows: 968.90 ± 321.30 m, 633.22 ± 184.72 m, and 588.8 ± 248.69 m, respectively, and the difference was statistically significant (CUMS: *p* < 0.05 and CUMS+FMT: *p* < 0.01, compared to the control group). Following saline administration, the total distance traveled by the rats in CUMS group was still significantly lower than that in the control group (*p* < 0.05). The total distance of rats in the CUMS+FMT group increased after FMT intervention compared to before FMT, although this difference was not statistically significant (*p* < 0.05; **Figure 2C**). There was a significant difference in the immobility time of rats in the three groups (*p* < 0.05 compared with the control group). The immobility time of rats was significantly higher in the CUMS group of rats gavaged with normal saline compared to rats in the control group (*p* < 0.01). The immobility time was decreased in the CUMS+FMT group compared to before FMT (*p* > 0.05; **Figure 2D**). After the rats were exposed to the CUMS methods, the upright number of times in the CUMS and CUMS+FMT groups differed significantly compared to the control group (*p* < 0.001 and *p* < 0.01, respectively). Following gavages, rats in the CUMS group showed significantly less upright number of times than those in the control group (*p* < 0.05), and the upright number of times in the CUMS+FMT group significantly increased after FMT (*p* < 0.05; **Figure 2E**).

#### Forced swimming test

The immobility time in the forced swimming test of the CUMS and CUMS+FMT groups significantly differed compared to the control group (*p* < 0.01 and *p* < 0.001, respectively). The immobility time in the forced swimming test in the CUMS group significantly increased after the rats were gavaged with normal saline (*p* < 0.001). Furthermore, the immobility time in the CUMS+FMT group significantly decreased after FMT compared to before FMT (*p* < 0.001; **Figure 2F**).

#### Glass bead discharge test

After FMT, the expulsion time of the glass beads was 238.30 ± 50.55 min, 315.00 ± 84.72 min, and 243.10 ± 54.89 min in the control, CUMS, and CUMS+FMT groups, respectively. The expulsion time of the glass beads was significantly longer in the CUMS group than in the control group (*p* < 0.05). Furthermore, the expulsion time was significantly shorter in the CUMS+FMT group after FMT (*p* < 0.05; **Figure 2G**).

### FMT has important effects on biochemical indices in the CUMS-induced rat model of depression

#### Immunohistochemical analysis of 5-HT, GABA, Glu, and BDNF levels

**Figure 3:**
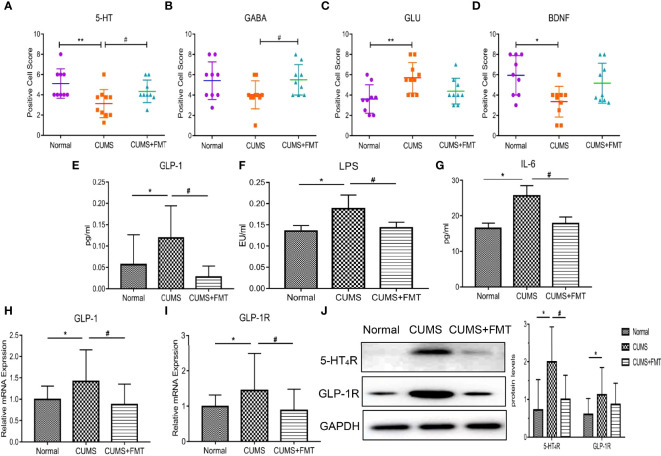
Changes in biochemical indices of each group. **(A–D)**, Expression of 5-HT, GABA, Glu, and BDNF in hippocampal tissues from each group; **(E–G)**, Expression of serum GLP-1, LPS, and IL-6 levels in each group; **(H–I)**, Expression of GLP-1 and GLP-1R in colonic tissue; **(J)**, Expression of 5-HT_4_R and GLP-1R. *p < 0.05, **p < 0.01 compared to the control group;**^#^***p* < 0.05 compared to the CUMS group. 5-HT, 5-hydroxytryptamine; 5-HT_4_R, 5-hydroxytryptamine 4-receptor; GABA, gamma-aminobutyric acid; Glu, glutamate; BDNF, brain derived neurotrophic factor; GLP-1, glucagon-like peptide-1; GLP-1R, glucagon-like peptide-1 receptor; LPS, lipopolysaccharide; IL-6, interleukin-6; CUMS, chronic unpredictable mild stress; FMT, fecal microbiota transplantation.

**Figure 4:**
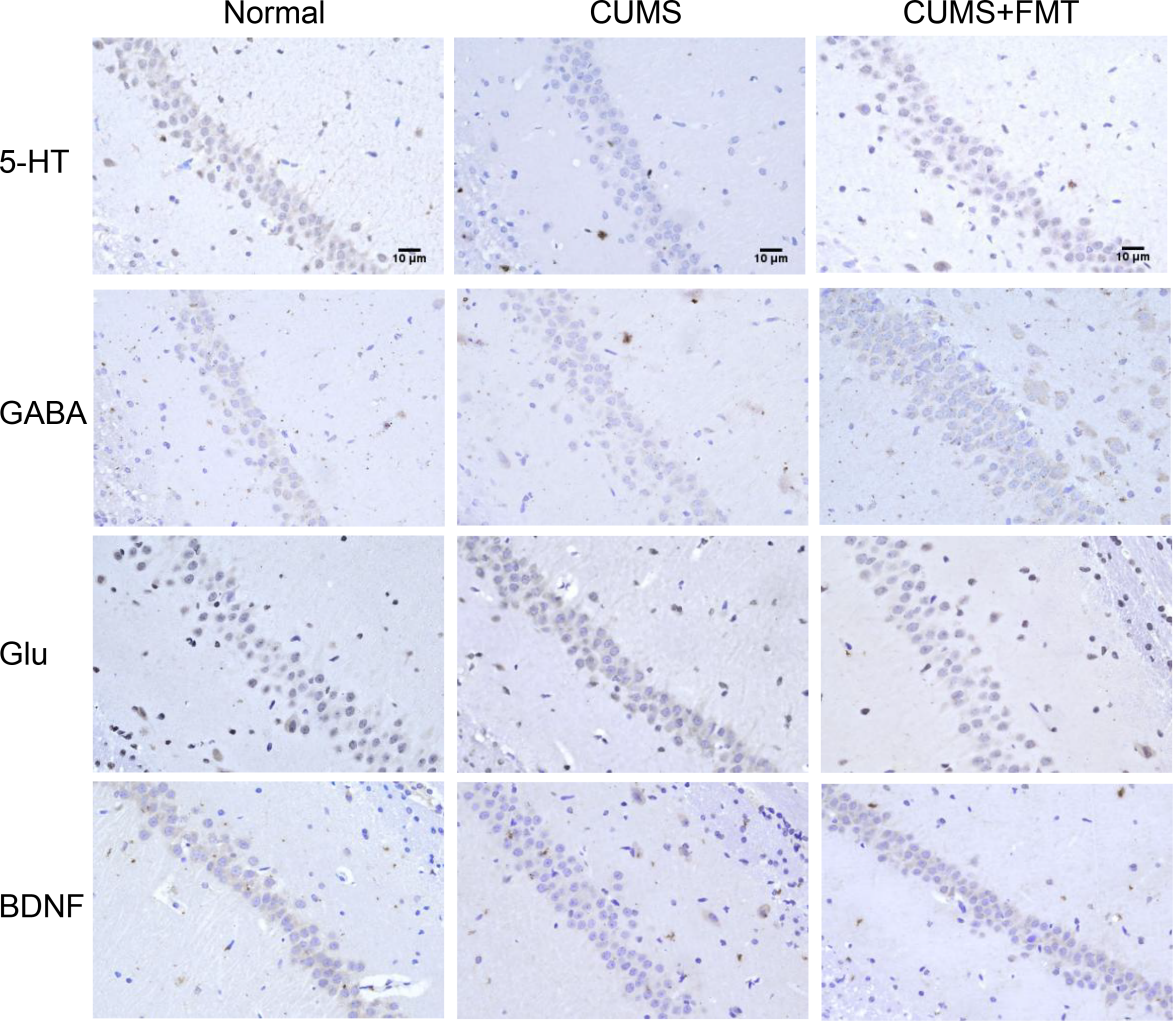
Levels of 5-HT, GABA, Glu, and BDNF in the hippocampus detected by immunohistochemistry (400× magnification). 5-HT, 5-hydroxytryptamine; GABA, gamma-aminobutyric acid; Glu, glutamate; BDNF, brain derived neurotrophic factor; CUMS, chronic unpredictable mild stress; FMT, fecal microbiota transplantation.

Results of the immunohistochemical analysis revealed that the levels of 5-HT, GABA, and BDNF in hippocampal tissues were significantly decreased in the CUMS group compared to the control group, while 5-HT and BDNF levels increased significantly in the CUMS+FMT group. The decreased expression of 5-HT and BDNF in the CUMS group was statistically significant (*p* < 0.01 and *p* < 0.05, respectively). The levels of 5-HT and GABA were significantly higher in the CUMS+FMT group compared to the CUMS group (*p* < 0.05), whereas the opposite effect was seen for levels of Glu (**Figures 3A–D** and **4**).

#### Serum GLP-1, LPS, and IL-6 levels detected by ELISA

The levels of GLP-1, LPS, and IL-6 were significantly increased in the CUMS group compared to the control group, whereas these levels were significantly decreased in the CUMS+FMT group compared to the CUMS group (**Figure 3E**, GLP-1 levels in the control, CUMS, and CUMS+FMT groups: 0.057 ± 0.067 pg/mL vs. 0.119 ± 0.071 pg/mL vs. 0.028 ± 0.024 pg/mL, *p* < 0.05; **Figure 3F**, LPS levels in the control, CUMS, and CUMS+FMT groups: 0.135 ± 0.013 EU/mL vs. 0.188 ± 0.031 EU/mL vs. 0.143 ± 0.013 EU/mL, *p* < 0.05; **Figure 3G**, IL-6 levels in the control, CUMS, and CUMS+FMT groups: 16.50 ± 1.36 pg/mL vs. 25.60 ± 2.73 pg/mL vs. 17.80 ± 1.78 pg/mL, *p* < 0.05).

#### *GLP-1* and *GLP-1R* expression levels in colonic tissues detected by PCR

PCR was used to detect levels of GLP-1 and GLP-1R expression in the three groups. The results revealed GLP-1 expression in the intestinal tissues of rats in the CUMS group were increased relative to the control, but the expression in the CUMS+FMT group was significantly lower than that in the CUMS group (**Figure 3H**: 0.995 ± 0.303 vs. 1.417 ± 0.720 vs. 0.876 ± 0.466 in the control, CUMS, and CUMS+FMT groups, respectively, *p* < 0.05). Similarly, GLP-1R expression showed the same patterns as GLP-1 (**Figure 3I**: 0.989 ± 0.318 vs. 1.445 ± 1.018 vs. 0.881 ± 0.584, *p* < 0.05).

#### Western blot analysis of GLP-1R and 5-HT_4_R protein expression in colonic tissues

Western blot analysis was used to detect the protein expression levels of 5-HT_4_R and GLP-1R in rat intestinal tissues from each group. The results showed 5-HT_4_R expression was increased in the intestinal tissues of the CUMS group compared to the control and CUMS+FMT groups. A similar pattern was observed for GLP-1R protein expression (**Figure 3J**).

#### Effect of FMT on intestinal microbiota in rat model of CUMS-indued depression

**Figure 5:**
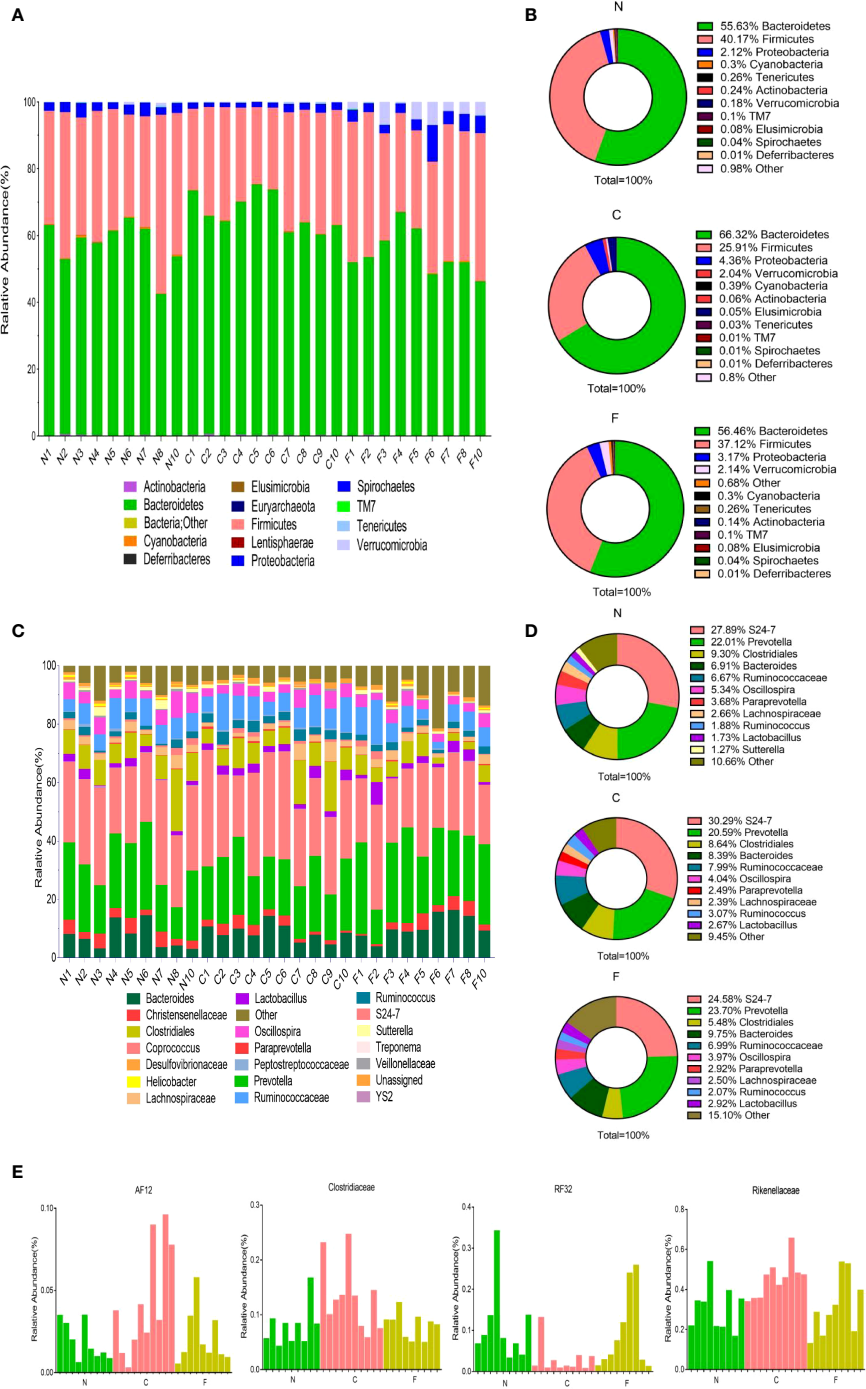
Results of 16s rDNA amplicon sequencing. **(A)**, Distribution of population composition by phylum rank; **(B)**, Pie chart of dominant populations by phylum rank; **(C)**, Distribution of population composition by genus rank; **(D)**, Pie chart of dominant populations by genus rank; **(E)**, Differences in selected populations of interest. N, Normal group; C, CUMS group; F, CUMS+ FMT group.

Fecal samples obtained from the control, CUMS, and CUMS+FMT groups were sequenced and analyzed. Of the 30 samples submitted for testing, two were not included for analysis due to quality issues of the extracted DNA. Sequencing of the fecal bacteria revealed significant differences among the three groups. With respect to phylum rank, the three groups of samples contain 14 phyla in total: Acidobacteria, Actinobacteria, Bacteroidetes, Cyanobacteria, Deferribacteres, Elusimicrobia, Euryarchaeota, Firmicutes, Lentisphaerae, Proteobacteria, Spirochaetes, Tenericutes, TM7, and Verrucomicrobia. **Figure 5A** shows the community composition of each sample at the phylum level, and **Figure 5B** shows the average abundance of each sample at the phylum level. Bacteroidetes, Firmicutes, and Proteobacteria were the dominant phyla in all three groups. The abundance of Cyanobacteria, Pericardium, and Actinomycetes was less than 0.1%. The abundance of Bacteroidetes and Proteobacteria was significantly higher in the CUMS group than in the control group, while the abundance of Bacteroidetes and Proteobacteria was significantly lower in the CUMS+FMT group compared to the control group. The abundance of Firmicutes decreased significantly in the CUMS group relative to the control group but increased significantly in the CUMS+FMT group. Verrucomicrobia was significantly more abundant in the CUMS group compared to the other two groups (*p* < 0.05).

At the family or genus levels (certain types can only be detected at the family level), a total of 84 distinct populations were detected in the three groups (**Figure 5C**). As shown in **Figure 5D**, S24-7 (Muribaculaceae), Prevotella, Clostridiales, and Bacteroides were the dominant populations in the three groups. The abundance of Ruminococcaceae was higher in the CUMS group relative to that in the control and CUMS+FMT groups (*p* > 0.05). The abundance of Ruminococcus was significantly higher in the CUMS+FMT group relative to the control group, but significantly lower than that in the CUMS group (p < 0.05). The abundance of Prevotella was significantly lower in the CUMS group than in the control group, and significantly higher in the CUMS+FMT group than in the control group (*p* < 0.05). In addition, based on the results of the sequencing data, certain specific populations were screened (AF12, Clostridiaceae, RF32, and Rikenellaceae), which revealed statistically significant changes (**Figure 5E**).

### Untargeted metabolomics results

#### OPLS-DA method to analyze fecal data

**Figure 6:**
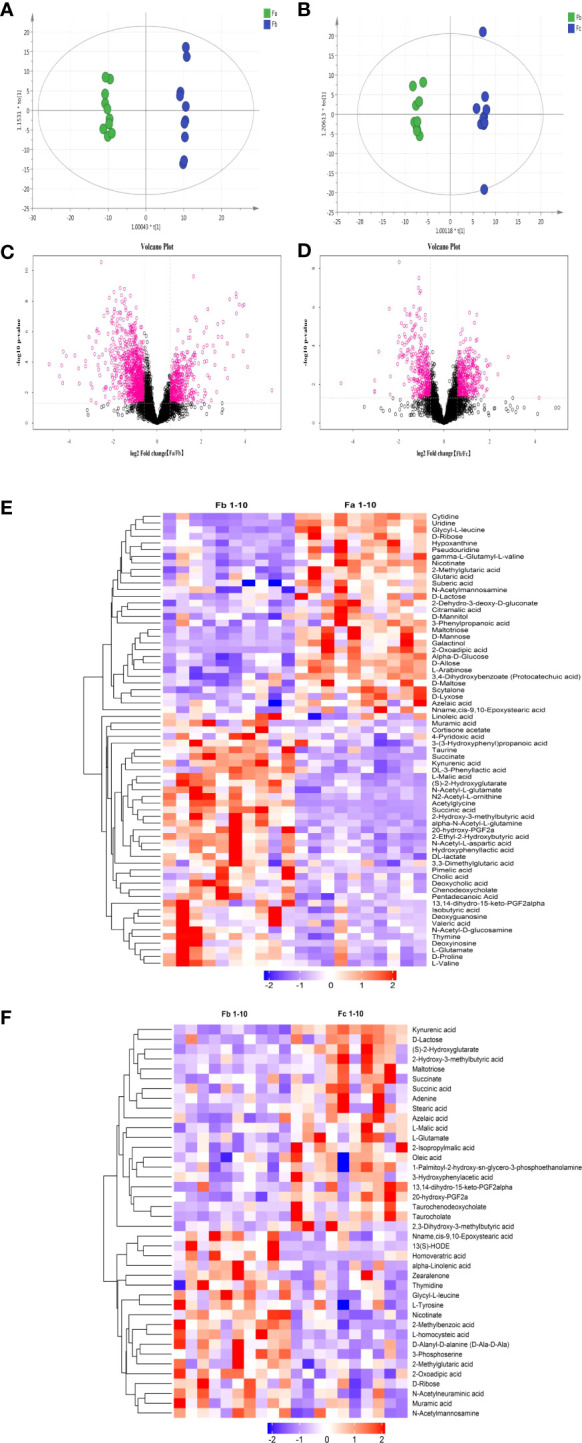
Results of untargeted metabolomics analysis. **(A)**, OPLS-DA analysis of fecal samples from the CUMS+FMT and CUMS groups before/after CUMS (Fa/Fb); **(B)**, OPLS-DA analysis of fecal samples from the CUMS+FMT group before/after FMT (Fb/Fc); **(C, D)** Volcano plot; **(C)**, Anion mode analysis of fecal samples from the CUMS+FMT group before/after CUMS (Fa/Fb); **(D)**, Anion mode analysis of fecal samples from the CUMS+FMT group before/after FMT(Fb/Fc). Red represents differentially expressed metabolites; **(E)**, Heat map analysis of fecal samples from CUMS+FMT group before/after CUMS (Fa/Fb); **(F)**, Heat map analysis of fecal samples from CUMS+FMT group before/after FMT (Fb/Fc); purple represents downregulation and red represents upregulation. OPLS-DA, Orthogonal Partial Least Square-Discriminate Analysis; Fa, fecal samples from the CUMS+FMT group before CUMS; Fb, fecal samples from the CUMS+FMT group after CUMS; Fc, fecal samples from the CUMS+FMT group after FMT; VIP, variable importance in projection; FC, fold change; CUMS, chronic unpredictable mild stress; FMT, fecal microbiota transplantation.

To determine the different metabolic indices of feces before and after CUMS and before and after FMT (or saline), the orthogonal partial least square-discriminate analysis (OPLS-DA) method was used to compare the metabolic status of rat fecal samples from the CUMS+FMT group before/after CUMS (Fa/Fb) and FMT (Fb/Fc). As shown in **Figures 6A, B**, there are obvious distinctions in the resulting data of each group before/after CUMS and FMT.

#### Screening potential differential metabolites

Univariate analysis was used to analyze differential metabolites between two groups of samples, including fold change (FC) analysis, t test, and volcano plot, which integrated the two analysis methods. **Figures 6C, D** illustrate the volcanic diagram of anion mode data before/after CUMS and before/after FMT (or saline), respectively. Each point in the figure represents a variable. The red points are metabolites with a FC > 1.5 and *p* < 0.05, i.e., red indicates the metabolites with differential expression. The heat maps (**Figures 6E, F**) showed the data separation more vividly before/after CUMS and FMT. It also shows changes in the metabolites with potential differences.

#### Determination of significantly different metabolites

According to the variable importance in projection values of the potential differential metabolites screened, and through mass charge ratio and comprehensive analysis of mass spectrometry data, a total of 13 metabolites with significantly differential expression before/after CUMS and FMT were identified. **Tables 2**, **3** list the potential biomarkers of FMT for the treatment of depression. Of these, 13,14-dihydro-15-keto-PGF2alpha, azelaic acid, glycyl-l-leucine, and muramic acid could not be determined by their metabolic pathways, which may be because these metabolites have not yet been included in the Kyoto Encyclopedia of Genes and Genomes database as they have been rarely studied to date.

**Table 2:** Differentially expressed metabolites and their variation trend according to variable importance in projection (VIP) values.

	Metabolites	Fa vs. Fb						Fb vs. Fc		
		VIP	FC		*p*-value	Trend	VIP	FC	*p*-value	Trend
1	(S)-2-Hydroxyglutarate	2.90		3.21	3.183E-05	**↑**	2.63	0.57	8.78E-04	**↓**
2	13,14-Dihydro-15-keto-PGF2alpha	1.65		2.18	2.107E-02	**↑**	1.81	0.54	4.83E-02	**↓**
3	2-Hydroxy-3-methylbutyric acid	5.53		4.42	9.580E-05	**↑**	4.44	0.50	4.81E-02	**↓**
4	2-Oxoadipic acid	7.61		0.22	1.664E-05	**↓**	4.29	1.66	2.51E-03	**↑**
5	Azelaic acid	1.89		0.85	2.420E-02	**↓**	3.17	0.77	1.33E-02	**↑**
6	d-Lactose	2.38		0.49	1.572E-02	**↓**	2.64	0.62	7.24E-03	**↑**
7	Glycyl-l-leucine	1.38		0.52	2.629E-06	**↓**	1.18	1.38	3.53E-03	**↑**
8	l-Glutamate	3.26		1.58	6.062E-03	**↑**	2.73	0.77	1.64E-02	**↓**
9	Maltotriose	4.00		0.27	1.564E-05	**↓**	1.10	0.56	3.78E-03	**↑**
10	Nicotinate	2.65		0.75	2.315E-04	**↑**	1.03	2.49	5.23E-04	**↓**
11	Succinic acid	19.53		17.10	1.823E-06	**↑**	14.80	0.51	3.60E-02	**↓**
12	Muramic acid	2.54		1.92	4.285E-02	**↑**	3.33	1.67	5.28E-02	**↓**
13	Succinate	7.55		3.25	2.365E-05	**↑**	5.31	0.67	6.53E-02	**↓**

VIP, variable importance in projection; Fa, fecal samples from the CUMS+FMT group before CUMS; Fb, fecal samples from the CUMS+FMT group after CUMS; Fc, fecal samples from the CUMS+FMT group after FMT; FC, fold change; CUMS, chronic unpredictable mild stress; FMT, fecal microbiota transplantation.

**Table 3:** Differentially expressed metabolites and their metabolic pathways according to variable importance in projection (VIP) values.

No.	Metabolites	Rest Time (min)	Mass Charge Ratio (m/z)	Metabolic Pathway
1	(S)-2-Hydroxyglutarate	720.98	147.03	Amino acid metabolism
2	13,14-Dihydro-15-keto-PGF2alpha	144.12	353.23	————
3	2-Hydroxy-3-methylbutyric acid	266.97	117.06	Amino acid metabolism
4	2-Oxoadipic acid	660.86	141.02	Amino acid metabolism
5	Azelaic acid	623.79	187.1	————
6	d-Lactose	592.71	341.11	Sugar/carbohydrate metabolism
7	Glycyl-l-leucine	510.14	187.11	————
8	l-Glutamate	722.65	146.05	GABAergic and Glutamergic metabolism
9	Maltotriose	792.28	503.16	Sugar/carbohydrate metabolism
10	Nicotinate	817.17	122.02	Cofactors and vitamin metabolism
11	Succinic acid	181.41	177.04	Glucagon signaling pathway
12	Muramic acid	463.86	310.11	————
13	Succinate	706.44	117.02	Glucagon signaling pathway

## Discussion

The gut microbiota affects various physiological functions in the host, and there is increasing evidence of a correlation between gut microbiota and depression. To explore the relationship between gut microbiota and depression and its related mechanisms, scientists have established many types of depression animal models and studied them extensively. Rao et al. (2021) found that FMT could ameliorate the gut microbial imbalance and intestinal barrier damage in rats with CUMS stress-induced depressive-like behavior. Liu et al. (2020) transplanted fecal microbiota obtained from patients with depression into germ-free rats through FMT technology, and rats that received FMT from individuals with depression then exhibited depression-like behavior, suggesting that microbiota may be involved in inducing depression-like behavior in rats. Interestingly, (Wang et al., 2021) found washed microbialtransplantation accelerated the recovery of abnormal changes caused by light-induced stress in tree shrews.

The CUMS model is a classic model of depression that is widely recognized by academic community. In this study, it was observed that after establishment of the model, the sucrose preference coefficient was significantly lower among rats in the CUMS and CUMS+FMT groups relative to the control group, and the sucrose preference coefficient was also significantly lower than the values measured before establishment of the model. The total distance traveled and the time spent upright during the open-field test decreased, while the time spent immobile in the open-field test and the forced swimming test increased, indicating a diminished interest in the outside world and the onset of depression in the model rats, suggesting the successful creation of the CUMS-induced depression model. The indicators of interest were reversed following treatment with FMT in the CUMS+FMT group, suggesting FMT may play an antidepressant role.

Gastrointestinal dysfunction is one of the main somatic symptoms during the onset of depression, and it is known to seriously impair patient quality of life. The central nervous system plays an important role in the regulation of gastrointestinal function. Stimulation of the internal and external environments can easily cause emotional changes in the body, which can also affect gastrointestinal function. While the food intake test can reflect the depressed state of rats, when combined with the glass bead test, it can also reflect the level of gastrointestinal motility. As the rats were in the acclimation stage, the food intake of the CUMS and CUMS+FMT groups continued to increase, but then decreased significantly in the same period compared with the corresponding levels in the control group. The food intake significantly increased after FMT in the CUMS+FMT group. The CUMS group only showed a slow increase, suggesting the appetite of the rats in the CUMS+FMT group improved after FMT. Similarly, the results of the glass bead test also indicated improved colonic function of rats in the CUMS+FMT group.

Many experiments have shown that the 5-HT transmitter system plays a vital role in the pathogenesis of depression (Seo et al., 2017; Pan et al., 2019). Gastrointestinal diseases in depressed patients can be improved to a certain extent after administering the 5-HT reuptake inhibitor (Cao, 2010). Meanwhile, gastrointestinal motility disorders are often associated with decreased 5-HT content, and gastrointestinal function can be improved by increasing the concentration of 5-HT *in vivo* (Israelyan et al., 2019). In a clinical study of patients with constipation, newly developed 5-HT_4_R agonists (ATI-7505 and TD-5108) were found to accelerate gastric emptying and colon motility, which resulted in an improved quality of life (Manabe et al., 2010; Goldberg et al., 2010). The 5-HT variation trend in the current study was consistent with that in the report mentioned above, which inferred a successful establishment of the depression model. The glass bead test suggested the colonic function of depressed rats was altered to slow motility, but the 5-HT_4_R levels in colonic tissues from the CUMS group increased, which may be the result of a compensatory mechanism.

BDNF is instrumental in the development and maturation of nerve cells. It has been found BDNF expression is downregulated in depressed patients (Serafini, 2012). Using animal models of depression, antidepressant effects have been produced after BDNF injection into the hippocampus (Castrén and Rantamäki, 2010). BDNF also contributes to intestinal motility. In healthy people, BDNF can accelerate intestinal emptying and increase the frequency of defecation (Coulie et al., 2000). BDNF can significantly enhance myoelectric activity of the intestinal smooth muscle, increase the peak of contractile amplitude of smooth muscle (Chai et al., 2003), and enhance the peristaltic reflex of isolated rat colons (Grider et al., 2006). Therefore, BDNF plays important roles to decrease depression and increase intestinal motility. In the current study, results of the immunohistochemical analysis revealed the levels of BDNF in the hippocampus of depressed rats were significantly reduced compared to control rats. Additionally, the level of BDNF was higher in rats in the CUMS+FMT group, suggesting BDNF contributed to the pathogenesis of depression and alteration of intestinal motility. FMT can increase BDNF expression, but the specific mechanism needs further study.

GABA and Glu are the main inhibitory and excitatory neurotransmitters, respectively, in the central nervous system and they function to maintain a balance of nerve excitation and avoid the occurrence of mood disorders. Studies have shown the GABA levels in the central nervous system are significantly decreased, while the Glu levels were significantly increased in patients with depression compared to those without (Pytka et al., 2016; Croarkin et al., 2013). Therefore, GABA- and Glu-associated pathways may also be common targets of depression (McMurray et al., 2018). Mitani et al. (2006) also found the serum levels of Glu in patients with depression were significantly increased, which was positively correlated with the severity of depressive symptoms. Following CUMS, Glu was upregulated and GABA was downregulated in the CUMS group, and both Glu and GABA levels were restored after FMT, indicating FMT had potential antidepressant effects.

Studies have shown that depression is associated with immune activation, and in recent years there has been an increasing focus on the important role of cytokines in the occurrence and development of depression (Pollak and Yirmiya, 2002; Nunes et al., 2002). Maes et al. (1995) suggested that increased IL-6 secretion, which is the basis of various acute phase reactions and immune responses, is essential for the occurrence and development of depression. Meanwhile, increased IL-6 can also lead to overactivation of the hypothalamic–pituitary-adrenal axis and 5-HT metabolic disorder, producing depression-like behaviors. Lipopolysaccharide (LPS) is considered a powerful pro-inflammatory mediator. Following an injection into mice with a low dose of LPS, the immune system rapidly activates, which was found to lead to depression-like behavior (O’Connor et al., 2009). Additional research has gradually revealed the antipsychotic effects of GLP-1 and its receptor agonists. Animal studies have shown that direct injection of GLP-1 into the central nervous system of rodents can increase anxiety-like behaviors (Gulec et al., 2010), and injection of GLP-1R agonists into the fourth ventricle or cerebral aqueduct of rats can significantly reduce feeding and body mass of rats (Hayes et al., 2016). Kanoski et al. (2011) has also shown that GLP-1 can suppress appetite and slow down gastric emptying through GLP-1R in the central nervous system. In the current study, IL-6, LPS, GLP-1, and GLP-1R were upregulated in the CUMS group and downregulated in the CUMS+FMT group compared to the corresponding levels in the control group, suggesting that FMT led to a reversal of CUMS-induced depressive symptoms, decreased appetite, and gastric motility disorders.

In our previous study (Cai et al., 2019), significant changes were found in the intestinal microbiota of patients with depression. In the study, depressive symptoms and gastrointestinal dysfunction such as abdominal distension, decreased appetite, and constipation were significantly improved following transplanting patients with intestinal microbiota from healthy donors. Based on these results, we continued the investigation in the present study using FMT and normal saline gavage for the CUMS+FMT and CUMS groups, respectively. Intestinal microbiota significantly differed between the CUMS+FMT and CUMS groups. The abundance of Bacteroidetes, Proteobacteria, Firmicutes, and Prevotella was significantly higher in the CUMS group, and the abundance of Firmicutes and Prevotella was significantly reduced in the CUMS group relative to the control group. This trend was reversed following FMT. Many studies (Turnbaugh et al., 2006; Ley et al., 2006) have shown that Bacteroidetes and Firmicutes can together promote the absorption or storage of energy by the host, and the increase of Firmicutes, the decrease of Bacteroidetes, or the increase in the proportion of Firmicutes/Bacteroidetes could help promote weight gain. Thus, the weight increase of rats in the CUMS+FMT group in the current study following FMT may also involve the participation of these two phyla. Proteobacteria have been shown to promote inflammation (Keshavarzian et al., 2015). The rise of Proteobacteria in depressed rats has verified the concept that “inflammatory mechanisms” mediate the occurrence of depression.

Kang et al. Found the proportion of Prevotella was reduced in autistic patients with accompanying gastrointestinal symptoms (Kang et al., 2013). Prevotella promotes the formation of short chain fatty acids (SCFAs) (Chen et al., 2017). SCFAs not only nourish intestinal microorganisms and intestinal cells, but they are also considered an important regulator of immunity and energy metabolism that also prevent the growth of bacterial pathogens (David et al., 2014). It was speculated that the transplantation of the intestinal microbiota from the control group to the CUMS+FMT group would enable the microbiota in the control group to colonize and propagate within the intestines of the CUMS+FMT group rats, optimizing the composition of intestinal microbiota among rats in the CUMS+FMT group, consequently improving depressive behavior and gastrointestinal function through a series of complex mechanisms and pathways mentioned above.

Metabonomics involves the study of the metabolic response to gene modification or pathophysiological stimulus response of the body, and it can be used to comprehensively understand chemical changes occurring in the body to explore underlying pathophysiological mechanisms (Nicholson and Wilson, 2003). In this research, 13 metabolites were identified that may be related to the pathophysiology of depression through the metabolomic analysis of fecal stool samples from the three experimental groups. The results revealed that these molecules were involved in the following pathways: amino acid metabolism, cofactor and vitamin metabolism, sugar/carbohydrate metabolism, GABA and Glu metabolism, and glucagon signaling pathway.

Amino acids are the precursor of CNS neurotransmitters and are important components of the immune system. Immune dysfunction is one of the important factors in the pathogenesis of depression. In addition, studies on patients with depression (Nicholson and Wilson, 2003) and animal models of depression (Ma et al., 2019) have shown that energy metabolism disorder is also related to the pathophysiological mechanism of depression. In the current study, (S)-2-hydroxy-2-hydroxyglutarate and 2-hydroxy-3methylbutyric acid, which are involved in amino acid metabolism, were decreased in depressed rats following CUMS, while 2-oxoadipic acid was increased following CUMS and reversed after FMT. d-Lactose, maltotriose, and nicotinate, which are involved in sugar/carbohydrate metabolism, also indirectly support the previous studies (Savignac et al., 2014; Ma et al., 2019). As mentioned above, the balance of GABA and Glu was associated with the occurrence of depression (Lener et al., 2017; Pham and Gardier, 2019). Studies have revealed changes in the concentration of GABA and Glu in the serum, plasma, and brain tissues of patients with mental disorders (Mathews et al., 2012; Duman et al., 2019). In this experiment, the l-glutamate level, which is mediated by GABA and Glu metabolites, was reduced in fecal samples from the CUMS group, which was consistent with the trend of biochemical tissue samples encountered in this study and results reported in the literature (Mathews et al., 2012). GLP-1 has antidepressant effects, inhibits gastric emptying, and reduces appetite. Succinic acid and succinate, which participate in the glucagon signaling pathway, were upregulated after CUMS and downregulated following FMT in rats.

The limitations of this study should be mentioned here. First, the incidence of depression has an obvious gender trend and is more common in females than in males; however, we used only male rats in the present research due to the lower success rate of building a depression model with both genders. Naturally, this may lead to some gender bias in our results. Further studies should explore the therapeutic mechanism of FMT in depressed female rats. Second, in our experiment, we observed that FMT produced an antidepressant effect on CUMS depression model rats. However, these mechanisms involved are broad and complex, and we did not deeply explore the relationships and inter-relationships between the these indicators. Future studies should further explore the antidepressant effects and seek the upstream targets involved.

## Conclusion

In this study, we successfully established a CUMS-induced depression model to explore the therapeutic effects and mechanisms of FMT in depression. FMT exerted antidepressant effects on CUMS-induced depression in rats, and the mechanism involved neural, inflammatory, endocrine pathways. Further studies are required to investigate their specific mechanisms.

## Data availability statement

The original contributions presented in the study are included in the article/supplementary material. Further inquiries can be directed to the corresponding author.

## Ethics statement

The animal study was reviewed and approved by The Committee for the Protection and Use of Laboratory Animals of the Central South University.

## Author contributions

TC performed the study, collected the data, performed the statistical analysis, and drafted the manuscript; S-PZ designed and performed the study; XS performed the study and revised the manuscript; L-ZY performed the statistical analysis and revised the manuscript; HH, BZ and S-LX performed the study; FW designed and organized the study, and supervised the development of the study, the writing process, and revised the manuscript. All authors have approved of the final version of the manuscript.

## Funding

This study was supported by the New Xiangya Talent Project of the Third Xiangya Hospital of Central South University (Grant no. 20180304); Hunan Provincial Natural Science Foundation of China (Grant no. 2020JJ4853); Hunan Provincial Clinical Medical Technology Innovation Guidance Project (Grant no. 2020SK53616); Scientific Research Project of Hunan Provincial Health Commission (Grant no. 202103032097); and Hunan Provincial Natural Science Foundation of China for Youths (2020JJ5609).

## Acknowledgments

We are deeply grateful to all the participants.

## Conflict of interest

The authors declare that the research was conducted in the absence of any commercial or financial relationships that could be construed as a potential conflict of interest.

## Publisher’s note

All claims expressed in this article are solely those of the authors and do not necessarily represent those of their affiliated organizations, or those of the publisher, the editors and the reviewers. Any product that may be evaluated in this article, or claim that may be made by its manufacturer, is not guaranteed or endorsed by the publisher.
